# Mother Knows Best: Dominant Females Determine Offspring Dispersal in Red Foxes (*Vulpes vulpes*)

**DOI:** 10.1371/journal.pone.0022145

**Published:** 2011-07-20

**Authors:** Helen M. Whiteside, Deborah A. Dawson, Carl D. Soulsbury, Stephen Harris

**Affiliations:** 1 School of Biological Sciences, University of Bristol, Bristol, United Kingdom; 2 Department of Animal and Plant Sciences, University of Sheffield, Sheffield, United Kingdom; Texas A&M University, United States of America

## Abstract

**Background:**

Relatedness between group members is central to understanding the causes of animal dispersal. In many group-living mammals this can be complicated as extra-pair copulations result in offspring having varying levels of relatedness to the dominant animals, leading to a potential conflict between male and female dominants over offspring dispersal strategies. To avoid resource competition and inbreeding, dominant males might be expected to evict unrelated males and related females, whereas the reverse strategy would be expected for dominant females.

**Methodology/Principal Findings:**

We used microsatellites and long-term data from an urban fox (*Vulpes vulpes*) population to compare dispersal strategies between offspring with intra- and extra-group fathers and mothers of differing social status in red foxes. Relatedness to the dominant male had no effect on dispersal in offspring of either sex, whereas there was a strong effect of relatedness to resident females on offspring dispersal independent of population density. Males with dominant mothers dispersed significantly more often than males with subordinate mothers, whereas dispersing females were significantly more likely to have subordinate mothers compared to philopatric females.

**Conclusions/Significance:**

This is the first study to demonstrate that relatedness to resident females is important in juvenile dispersal in group-living mammals. Male dispersal may be driven by inbreeding avoidance, whereas female dispersal appears to be influenced by the fitness advantages associated with residing with the same-sex dominant parent. Selection pressure for paternal influence on offspring dispersal is low due to the limited costs associated with retaining unrelated males and the need for alternative inbreeding avoidance mechanisms between the dominant male and his female offspring. These findings have important implications for the evolution of dispersal and group living in social mammals, and our understanding of a key biological process.

## Introduction

Dispersal is a fundamental biological process. Despite many studies into its underlying causes [1]–[3], little is known about genetic influences. At the population level, dispersal is thought to be a principal method of inbreeding avoidance [3]. Whilst an underlying assumption of this theory, hitherto no one has identified a role of intra-group relatedness on dispersal. Most canids are socially monogamous, but extra-pair copulations are common [4]–[6] and polygynandry creates social groups with juveniles of varying degrees of relatedness. Here, we tested whether relatedness to dominants determines offspring dispersal patterns in the red fox (*Vulpes vulpes*).

In group-living carnivores, dominant animals tolerate the presence of subordinates provided the reproductive and/or survival benefits outweigh the intra-specific costs [7]. Red fox social groups may contain additional subordinate adults, either offspring from previous years or individuals that have dispersed into the group [8]. Extra-pair copulations, and opportunistic subordinate matings, result in offspring with varied degrees of relatedness to the dominant pair [6]. Variation in relatedness is important in the evolution of dispersal, as kin competition and inbreeding avoidance are believed to be ultimate causes of dispersal [3].

In the absence of any fitness advantage for a dominant male, unrelated subordinate males might be expected to be forcefully evicted since they may compete with related males for resources, mates and future dominance, reducing the dominant male's indirect fitness. Forced dispersal of unrelated males would also reduce future conflicts between related and unrelated offspring e.g. over territory inheritance. Since dominant male foxes mate with resident subordinate females [6], the potential for inbreeding with related female offspring is high. If the costs of inbreeding outweigh the costs of dispersal, female offspring related to the dominant male should disperse whereas unrelated females, fathered through extra-group copulations, would be expected to be philopatric, providing future unrelated subordinate mating opportunities. However, a serious weakness in this argument is that juvenile dispersal in foxes is male biased [9].

If relatedness to dominant females is important, there is a potential conflict between dominant males and females if they parent the same offspring. Related males may disperse, avoiding inbreeding with dominant females, whereas unrelated males may disperse, avoiding resource competition with dominant males. Dominant females can potentially increase their fitness by allowing related females to remain and provide alloparental care to future offspring, but reduce intra-specific resource competition by evicting unrelated females.

Our aim was to determine whether juvenile dispersal is influenced by their relatedness to resident dominants and, if so, understand how the conflict between the dominant pair over offspring dispersal is resolved. We compared dispersal strategies of direct descendants of the dominants (related offspring) and cubs parented by subordinate females or extra-group males (unrelated offspring) using data from a long-term capture-mark-recapture study on red foxes to test three contrasting hypotheses: (i) paternal social status affects offspring dispersal; (ii) maternal social status affects offspring dispersal; and (iii) extra-group paternity affects offspring dispersal. We predicted that unrelated male offspring and related female offspring would disperse if relatedness to the dominant male was the major influence, whereas related male offspring and unrelated female offspring would disperse if relatedness to the dominant female had a stronger effect.

## Methods

### Study site and study animals

The study was undertaken in north-west Bristol, UK: this urban fox population has been studied continuously for over 30 years. Population density varied widely over the study period due to a sarcoptic mange (*Sarcoptes scabiei*) epizooty from 1994, which eliminated foxes on the study area in 1996 [10]; recovery thereafter has been slow [11]. Therefore we divided the samples into two time periods: (i) cohorts of cubs born in 2004–2009 when population density was 5.5–25.5 adults per km^2^, referred to as the high-density sample [12]. To include putative parents, we genotyped 410 foxes (212 males, 198 females) captured between 2002–2009; these comprised 92% of captures; and (ii) cohorts of cubs born in 1999–2003 when fox population density was 4.0–5.5 adults per km^2^, referred to as the low-density sample [12]. To include putative parents, we genotyped 146 foxes (76 males, 70 females) captured between 1998–2003; these comprised 95% of captures. Forty-four animals were included in both time periods, as cubs born during the low-density period were candidate parents during the high-density period.

Foxes were captured either by netting from den sites or in baited box traps set in residential gardens [13]. They were handled manually or anaesthetized with an intramuscular injection of ketamine hydrochloride (Vetalar 100 mg/ml solution; Pfizer Limited, Sandwich, Kent, UK), sexed and marked with plastic ear tags (Rototags, Dalton Supplies Ltd., Henley-on-Thames, Oxfordshire, UK); selected animals were also fitted with a radio collar. Three age classes were recognized based on body size and incisor wear [14]: cubs were <6 months old, subadults 6–12 months, and adults >12 months. All animals were assumed to have been born on April 1^st^ each year [9]. Animal capture and handling procedures conformed to guidelines by the American Society of Mammalogists [15], were approved by the University of Bristol's ethics committee and were carried out under the Animals (Scientific Procedures) Act 1986 license number PPL3002434.

Foxes were assigned to social groups using capture-mark-recapture data and radio telemetry [16]. Any individual found on the same territory for two successive captures was assumed to have been resident on that territory, and thus a member of that social group, for the intervening period [6]. Dominant animals were those that elicited submissive behavior during interactions with all other same-sex group members and, for females, the individual most closely associated with the cubs when only a single litter was present [6]. Only animals first captured as cubs were classified as dispersers or philopatric and all cubs were assumed to have been captured on their natal territory. Dispersers were any subadult or adult recovered dead or recaptured one or more territory diameters away from the point of first capture. Philopatric animals were any adult recovered dead or recaptured less than one territory diameter away from the point of first capture: subadult recaptures were not classified as philopatric since this is the age-class when dispersal occurs [9]. Territory diameter was calculated separately for each year to take account of changes in fox population density.

### Laboratory work

Genomic DNA was extracted from ear tissue ejected during marking, using an ammonium acetate precipitation method [17], [18]. Twenty-four markers (Table 1) were tested for establishing parentage in the high-density sample. All loci were unique based on a BLAST comparison of their sequences using a stand-alone BLAST and utilizing the GenBank nucleotide database (nr). The genome locations of the loci in the dog genome [19] were obtained based on sequence homology to check for potential physical linkage between loci or if any of the loci were located on the sex chromosomes. Duplex touchdown PCR (conditions below) were carried out on 24 unrelated individuals (12 males and 12 females) from the Bristol population to assess the loci. Fifteen markers, 11 from domestic dogs [20] and 4 from red foxes ([21]; Table 1) were selected for parentage analysis because they showed robust amplification, were autosomal (based on genotyping of known sexes and sequence homology to the assembled dog genome), had a low estimated frequency of null alleles (<0.1), had low levels of allelic drop out, lacked linkage disequilibrium and adhered to Hardy-Weinberg equilibrium (p>0.05). Observed and expected heterozygosity and estimated null allele frequencies were calculated using CERVUS version 3.0 [22]. Hardy-Weinberg equilibrium and linkage disequilibrium was calculated using GENEPOP version 4.0.10 [23]. PEDANT version 1.3 [24] was used to estimated allelic dropout rate per allele and the false allele rate per genotype.

**Table 1:** Details of microsatellite markers used to genotype Bristol's fox population and markers rejected for genotyping.

Marker	Sequence accession number	Locus source species	Forward primer sequence	Reverse primer sequence
1DGN14	NW_876272	*Canis familiaris*	TCACACAAAGTGGGTAAGATGG	GATTATGGTGCTATCCCTCTGG
1DGN3	NW_876269	*Canis familiaris*	TTTTTTCTGTAAACCTAAAGCTGC	GGAAAGGTACAGGCATGTAGTTGG
1FH2017^b^ ^c^ ^d^	NW_876259	*Canis familiaris*	AGCCTCTATAATCACGTGAGCC	CCCAGTACCACCTTCAGGAA
1FH2131^a^	NW_876308	*Canis familiaris*	ATGAAGCCTCACGCCAAG	TGATCACACTCATCTCCCCA
1FH2174	NW_876323	*Canis familiaris*	CACCTGTTCTCATAGAATGCAG	AAGTCTCGCCTCGGGGTC
1FH2201^a^	NW_876323	*Canis familiaris*	ATCAACAATGCATGCCACAT	GAGAACAAATAAATGCAAGCCC
1FH2226^b^ ^d^	NW_876323	*Canis familiaris*	GGACTACCCCATTGCATTTG	GAATCGAGTCCCATATCGGG
1FH2281	NW_876277	*Canis familiaris*	TGCTGGCACGTATACCAAGA	AGTGTGATGCAGAGGTTCCC
1FH2289^a^	NW_876284	*Canis familiaris*	CATGGTCTCAGGATCCTAGGA	CTAAGCATTCTCTCTGATGGTCTT
1FH2309	NW_876270	*Canis familiaris*	GACTGAGTTCTTTCAGCACAGTG	GGCAGCCTTATTATTCATGGA
1FH2316	NW_876307	*Canis familiaris*	AAATGGCCTGACGAATATGC	GTGCCATGGCATATGGTAAA
1FH2348^b^ ^d^	NW_876256	*Canis familiaris*	GCATGCAAAGGTGTTAATTGG	ACACAAGGAAGCTTTGGGG
1FH2541	NW_876307	*Canis familiaris*	CGTATGAGTTGGTATAATCTCAGG	TGCTTTTCACCTCCCTCTTG
1FH2658	NW_876258	*Canis familiaris*	TCTTAGAAATTGCTGGTGGG	TAAGAAACTGCCAGTCTGTGG
1REN161A12	NW_876308	*Canis familiaris*	GCCAAATGTCTCAGATGGGT	TGTCCACAGCTCATGAAAGG
1REN162B09	NW_876269	*Canis familiaris*	CAAACTTGACAGTCTTTTCAGGA	GCATTCAAGATGCACCAATG
1REN69B24	NW_876323	*Canis familiaris*	TGTAGGGCAGTGAATAAAAG	GCCTGGCTCAAGCTCACAAGT
1V142	DQ118707	*Vulpes vulpes*	AAGCAGATCCTAGAGCAGCA	CCCCACAGTTTAGAAATATCTGC
1V374	DQ118709	*Vulpes vulpes*	TACACACAGGAAGTAATGGGG	GACAGAAAGACAGAAGGCTTAG
1V402^c^ ^d^	DQ118717	*Vulpes vulpes*	GGGTAATTCATCCAGTGCCTT	TATGCAAACATGCAAACATGC
1V468	DQ118718	*Vulpes vulpes*	TCTCCCACCCAAATCTCTTG	GCATTCAAGATGCACCAATG
1V502^b^ ^c^ ^d^	DQ118722	*Vulpes vulpes*	ACCCAAGTGTCCTCCATAGAT	TGGCCAAGTACTCTTCCACT
1V602	DQ118723	*Vulpes vulpes*	CAGCCTGGACTACAATTCTCTTT	CCCCAAGTCTTTTGTCCAGA
1V622^a^	DQ8730	*Vulpes vulpes*	TTTTTTGAAAAGCACACCC	TGCTTTGTGTATCTTTTCTTTC
2aht130	NW_876266	*Canis familiaris*	CCTCTCCTGGTAATTGCTGC	TGGAACACTGGTCCCCAG
2c2001	L78573	*Canis familiaris*	TCCTCCTCTTCTTTCCATTGG	TGAACAGAGTTAAGGATAGACACG
2c2006	L78577	*Canis familiaris*	TGGGGGCGTTAAGAGTAATG	CTAGGCCTAAACCCCTGAGC
2c2010	L78579	*Canis familiaris*	AAATGGAACAGTTGAGCATGC	CCCCTTACAGCTTCATTTTCC
2c2017	L78583	*Canis familiaris*	AGCCTCTATAATCACGTGAGCC	CCCAGTACCACCTTCAGGAA
2c2054	L78589	*Canis familiaris*	GCCTTATTCATTGCAGTTAGGG	ATGCTGAGTTTTGAACTTTCCC
2c2079	L78596	*Canis familiaris*	CAGCCGAGCACATGGTTT	ATTGATTCTGATATGCCCAGC
2c2088	L78599	*Canis familiaris*	CCCTCTGCCTACATCTCTGC	TAGGGCATGCATATAACCAGC
2ucb466	L27191	*Canis familiaris*	TCTGGATTGTGGTCACAACC	ACTGGACACTTCTTTTCAGACG
2ucb646	L29310	*Canis familiaris*	TGGGATTCCAAAATGTTTTT	TCCCAGGATTAAGTCCCACA

1 markers tested for establishing parentage in the high-density sample.

2 markers used for establishing parentage in the low-density sample.

a marker not used in genotyping due to poor amplification.

b marker not used in genotyping due to high frequency of nulls alleles.

c marker not used in genotyping due to linkage disequilibrium.

d marker not used in genotyping due to violations of Hardy Weinberg equilibrium.

The 15 selected loci were amplified in 4 multiplex sets (Table 2) using the Qiagen multiplex kit (Qiagen, Hilden, Germany) following Kenta *et al.* [25]. Each 2-µl multiplex reaction contained 50 ng genomic DNA, 1 µl Qiagen master mix and 1 µl primer mix (where all primers were at 0.2 µM). Due to differences in primer annealing temperatures, a touchdown program (65-55°C) was used for PCR amplification using a DNA Engine Tetrad thermocycler (MJ Research, Waltham, USA) with the following program: 3 minutes at 95°C followed by cycles of 30 seconds at 94°C, 90 seconds at 65-55°C (annealing temperature dropped by 1°C every cycle with 35 cycles at 55°C) and 60 seconds at 72°C, followed by a final extension stage of 30 minutes at 60°C. A negative PCR control (containing no DNA) was used to check for PCR contamination and individuals of known genotype were included on each plate to enable consistent allele size scoring. PCR products were separated using a 48-well capillary ABI 3730 automated DNA Sequencer (Applied Biosystems, Warrington, UK) with ABI ROX500 size standard (Applied Biosystems). Data were analyzed using GENEMAPPER version 3.7 (Applied Biosystems). All 410 foxes were examined to assess if any of the loci were sex-linked.

**Table 2:** Characterisation of 15 microsatellite markers in 24 unrelated foxes from Bristol's high-density population.

Marker	Multiplex set	N	Florescent label	Alleles observed	Allele size range (bp)	HWE p-value	Non-exclusion probability parent pair	Estimated frequency of null alleles	HObs	HExp
FH2541	1	23	HEX	8	170–206	0.5691	0.2122	+0.0530	0.783	0.814
REN69B24	1	20	6-FAM	7	228–280	0.7865	0.2293	−0.0391	0.750	0.771
REN161A12	1	22	HEX	5	295–303	0.8629	0.4392	−0.0680	0.682	0.608
V374	1	23	HEX	4	110–116	0.6962	0.3335	−0.0647	0.739	0.738
V468	1	21	6-FAM	5	83–93	0.6550	0.2844	−0.0609	0.762	0.736
V602	1	23	6-FAM	5	137–162	0.1400	0.3753	+0.0281	0.609	0.663
DGN3	2	22	6-FAM	9	192–250	0.7768	0.1517	−0.0177	0.864	0.854
DGN14	2	18	HEX	7	224–250	0.7546	0.2102	+0.0105	0.722	0.811
REN162B09	2	24	HEX	2	190–194	1.0000	0.6793	−0.0237	0.500	0.507
V142	2	23	HEX	6	131–143	0.0906	0.2945	+0.0634	0.652	0.727
FH2174	3	20	HEX	9	232–276	0.6574	0.1690	+0.0102	0.850	0.831
FH2658	3	13	6-FAM	14	352–449	0.2187	0.0677	+0.0682	0.846	0.938
FH2281	4	23	6-FAM	9	429–465	0.8526	0.1571	−0.0777	0.957	0.849
FH2309	4	23	6-FAM	5	350–370	0.2808	0.2980	+0.0754	0.652	0.766
FH2316	4	21	HEX	11	282–368	0.9346	0.1232	−0.0582	1.000	0.878

The low-density sample was genotyped at 10 loci (Table 1) as part of a previous study [26]. Genotypes were assigned using a Megabase™ 1000 capillary sequencer (Amersham Pharmacia Biotech Ltd, Buckinghamshire, UK) with fragment sizing and allele calling completed with the associated software GENETIC PROFILER v.1.5. To minimize genotyping errors, allele scoring was undertaken independently by 2 people. The DNA extraction method, microsatellite loci identities, PCR reaction conditions, genotyping methods, heterozygosities and Hardy-Weinberg probabilities are described in Soulsbury *et al.* [26].

### Parentage analysis

Since foxes breed during their first year [27], candidate mothers were females aged >9 months known or believed to be present on the cubs' territory from January to October i.e. from the onset of the mating season to the onset of dispersal by that cohort of cubs. Candidate fathers were all males present on the study area during the mating season i.e. the January and February prior to the cubs' birth. Analysis was carried out separately for each cohort of cubs, and the two sets of microsatellite genotyping data were run independently.

We adopted a likelihood method to assign parentage, using the software COLONY version 2.0 [28]. Mating systems were defined as polygynandrous without inbreeding for both sexes. No maximum number of siblings or known relatives were assumed. The proportion of candidate parents sampled was estimated at 70% using capture records [13]. However, since trapping rates could vary with population density, we used the 2009 cohort cubs to compare the effects of a variety of trapping rates (10%, 25%, 70%, 90%). PEDANT-estimated allelic dropout rate of each locus and mean genotyping error from regenotyping were incorporated into the analysis. For consistency, parentage analysis was rerun for the low-density sample using COLONY and the results compared with the previous study [12]. Three problematic loci from the low-density sample, c2006, c2088 and aht130, were not included due to poor scoring, high allelic dropout and/or significant departures from Hardy-Weinberg equilibrium [26]. Re-genotyping data were not available for this sample so the same error rate was assumed. Full likelihood analyses were run 3 times to ensure consistent parentage assignments.

### Statistical analysis

The effect of relatedness to dominants on offspring dispersal was assessed separately for each sex using two-way Fisher's exact tests (SPSS version 16, IBM Corporation 2010, Chicago, USA) for all samples combined and for high-density samples separately. Low-density samples could not be analyzed separately due to sample size. Relatedness to dominant males was tested by comparing offspring dispersal strategies between dominant fathers and extra-group fathers. Relatedness to dominant females was tested by comparisons of offspring dispersal strategies between dominants and subordinates. Means are given ± SE.

## Results

### Microsatellite data

For the high-density population, re-genotyping of 23 individuals (5% of samples) provided a genotype error rate of 3.68%. All 15 microsatellite loci used to genotype the high-density samples were polymorphic and easily scorable. Locations of the loci on the dog genome were assigned using the ENSEMBL web interface (http://www.ensembl.org/Canis_familiaris/blastview) when the E-value was stronger than E-05. The following sequences were utilized to assign genome locations: (i) the amplified region; (ii) the EMBL sequence; and (iii) a 12,180 bp fragment of the sequence surrounding the amplified region. Fourteen loci were assigned to 8 different autosomes based on sequence homology. When multiple sequence sources were available and used for the same locus, all assigned locations were consistent. One fox sequence, V602 (EMBL accession number DQ118723), could not be assigned a location.

The 212 males and 198 females were all genotyped at each locus. No loci were X-linked based on genotyping, as all loci were heterozygous in a proportion of both males and females: X-linked loci would appear homozygous in all males (XY). Females (XX) amplified at all loci, so no loci were Y-linked. The average number of alleles per locus was 7.13, with a mean observed heterozygosity of 0.76±0.07 and mean expected heterozygosity of 0.77±0.05. Estimated mean allelic dropout rate per allele was 0.06±0.03. There was a mean false allele rate per genotype of 0.03±0.01. No pairs of loci were found to display linkage disequilibrium following a sequential Bonferroni correction [29] and no locus deviated from Hardy-Weinberg equilibrium (Table 2). However, FH21746 and REN69B24 were mapped closely on dog chromosome 7. To ensure any potential physical linkage between these loci did not affect parentage assignments, cubs from 2009 were analyzed three times: (i) with both markers; (ii) excluding FH2174; and (iii) excluding REN69B24. No differences in parentage assignments between the three analyses were found and the results adhered to what was expected from behavioral observations. Similarly, there were no differences in 2009 parentage assignments between simulations run with varying sampling proportions of candidate parents i.e. trapping rates of 10%, 25%, 70% and 90%. Thus, 70% was used for each cohort. The combined non-exclusion probability for the parent pair at all 15 loci, calculated using CERVUS, was 4.33e^−10^.

For the low-density sample, the average number of alleles per locus was 7.90, with a mean observed heterozygosity of 0.60±0.06 and mean expected heterozygosity of 0.72±0.04 [26]. Markers were tested for violations of Hardy-Weinberg equilibrium [12], [26] and linkage disequilibrium [12]. Parentage was assigned using CERVUS v. 2.0 and a decision matrix [12]. Mean polymorphic information content was 0.687; exclusionary power of the first parent was 0.989 and 0.999 for the second parent [12]. We also examined genotypes for sex linkage. All loci were heterozygous in a proportion of both males and females. Females amplified at all loci, so no loci were Y-linked. For the loci reanalyzed using COLONY, no inconsistencies were found between methods.

### Effect of parentage on offspring dispersal

**Figure 1:**
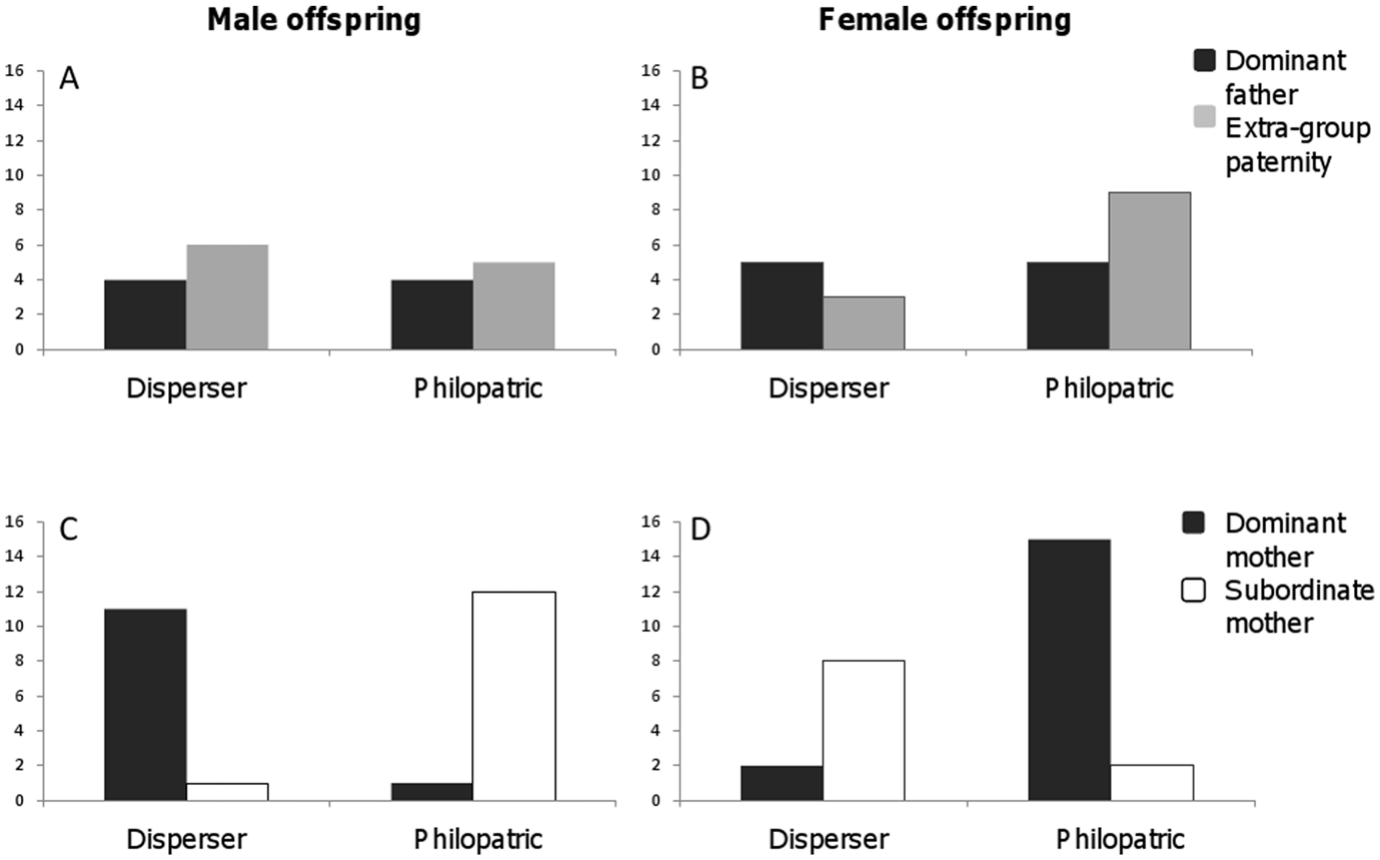
Frequency of dispersing and philopatric red fox offspring from parents of differing social status. Paternal group association is shown for male offspring (A) and female offspring (B). Maternal social status is shown for male offspring (C) and female offspring (D). Maternal social status had a sex-dependent influence on offspring dispersal, whereas the father's social group had no effect.

Only foxes with known dispersal status and assigned parents with established social status and/or extra-group paternity could be analyzed; thus 24% of all cubs captured between 1998 and 2009 were used (Table 3). Paternity did not affect male (Fig. 1A, p = 1, N = 19) or female (Fig. 1B, p = 0.378, N = 22) offspring dispersal, whereas maternity did. Males with dominant mothers dispersed significantly more often than males with subordinate mothers (Fig. 1C, p<0.001, N = 25). In contrast, dispersing females were significantly more likely to have subordinate mothers compared to philopatric females (Fig. 1D, p<0.001, N = 27). Analyzed separately, the high-density samples showed the same pattern. Paternity did not have an effect on offspring dispersal for males (p = 0.070, N = 13) or females (p = 0.999, N = 17), whereas maternity significantly affected male (p = 0.004, N = 18) and female (p<0.001, N = 22) dispersal. Low-density samples showed a similar trend but samples sizes were too low (N = 24) for statistical analysis. Only two cubs with known dispersal status were assigned subordinate fathers. They followed the expected dispersal strategies if maternal social status affects juvenile dispersal: a male with a dominant mother dispersed, a male with a subordinate mother was philopatric.

**Table 3:** Numbers of foxes genotyped.

	High density		Low density	
	Males	Females	Males	Females
Total number of individuals genotyped	212	198	76	70
Total number of cubs genotyped	85	83	46	36
Cubs with determined paternity only	4	5	2	3
Cubs with determined maternity only	36	36	7	7
Cubs with determined paternity and maternity	30	29	13	11
Cubs with known dispersal and paternal group association only	2	3	2	0
Cubs with known dispersal and maternal social status only	5	7	3	1
Cubs with known dispersal and both paternal group association and maternal social status	12	15	5	4

### Data quality

Difficulties with identifying dispersal strategy, parentage, parental social status and group association potentially created biased sampling. During 2002–2009, 52% of captured cubs were male, 48% female. It is unlikely therefore that there was a bias due to sex differences in births or trapping rates. Similarly, there was no difference between intra- (49%) and extra-group (51%) paternity assignments. This was particularly important as the trapping regime and identification of candidate fathers could have lead to an underestimate of extra-group paternity. Whilst only 16 (8%) of the cubs assigned paternity had subordinate fathers, dominant males monopolize intra-group breeding, so it is unlikely that the low frequency of subordinate fathers is a result of sampling bias. We identified four mixed paternity litters and subordinate mothers were present in each year at all densities. Thus, we believe that our sample is a true representation of the population.

## Discussion

We found a strong effect of maternal social status on dispersal in both male and female offspring, which was not affected by population density. In contrast, relatedness to dominant males did not affect dispersal in offspring of either sex.

### Dominant males and offspring dispersal

There are a number of reasons why relatedness to the dominant male may not influence dispersal in red foxes. Unrelated subordinate males and dominant males are not in direct reproductive competition because dominant males monopolize breeding with resident females, whereas subordinates seek matings on other territories [6]. It is unlikely that unrelated males compete for food, as this is available in excess [30]. So unrelated philopatric male offspring impose little cost on the dominant male, but equally the advantages of retaining male offspring are limited. Alloparental care by subordinates provides minimal fitness benefits to the dominant pair [16]. Moreover, at high population density few philopatric males gain dominance in their natal group [12], so there is a low chance of inheriting the territory. Therefore there is little selection pressure on dominant males to influence male dispersal.

Whilst we expected female offspring sired by dominant males to avoid inbreeding through dispersal, we found no such effect. This may be because dominant males tend to be unrelated to other adults in the social group due to extra-pair copulations and polygynous group reproductive output [12]. Moreover, interannual turnover of dominant males was high during periods of low population density [12], so there was limited risk of a dominant male mating with his philopatric female offspring. In addition, red fox dispersal is sex biased, with males leaving more frequently and travelling further than females, which generally move into adjacent social groups [9]. This creates high levels of inter-group relatedness across adjacent territories [12], and may explain why dominant males travel up to 2.7 territory diameters in search of extra-pair copulations [6]. Consequently, dispersal is an inadequate mechanism of inbreeding avoidance between dominant males and their female offspring, and so there is little selection pressure on dominant males to influence female offspring dispersal.

### Dominant females and offspring dispersal

Numerous studies have suggested male-biased dispersal patterns evolved as an inbreeding avoidance mechanism [3]. The majority of within-group matings in red foxes are confined to the dominant male, with dominant females breeding at every opportunity, and subordinate females breeding at 56% of opportunities [6]. This creates a differential risk of inbreeding between mother and male offspring, dependent upon relatedness to the dominant female. We propose that males from dominant females have a higher probability of breeding with their mothers, and so disperse to avoid future inbreeding costs. In contrast, males with subordinate mothers adopt philopatry, as the costs of dispersal outweigh any potential risks of inbreeding.

This assumes that foxes disperse voluntarily. In some mammals the presence of the opposite sex parent or kin is sufficient stimulus to cause offspring dispersal [3], [31], although many species show increased aggressive behavior during the dispersal period, leading to the forced eviction of selected group members. If male red foxes are selectively evicted by their mothers, this implies the existence of kin recognition. Whilst widely reported in vertebrates [32], it is unknown whether foxes can recognize their own sub-adult offspring. However, if they can, the costs of dispersal could be avoided through alternative behavioral mechanisms of inbreeding avoidance, such as refusal to mate. Furthermore, in canid populations with limited dispersal, extra-pair copulations are highly efficient mechanisms of inbreeding avoidance [4]. Thus more data are needed on the role of agonistic interactions between male offspring and their mothers to conclude that inbreeding avoidance is the true explanation for the effect of relatedness to the dominant female on male dispersal.

Females with subordinate mothers dispersed more frequently than those with dominant mothers. Since dominants breed more frequently, there is a higher probability that the following year's cubs will be more closely related to the dominant's offspring from the current year than offspring from subordinates [6]. Furthermore, philopatric females reproduced significantly more than dispersers because dispersing females often missed their first breeding opportunity [27]. So philopatric female offspring related to the dominant female avoid the costs of dispersal while gaining indirect fitness benefits through alloparental care [33]. Moreover, by retaining her same-sex offspring, a dominant female has a higher probability of one of them inheriting the territory [16], increasing both her own and one of her female offspring's fitness. In contrast, retention of unrelated subordinate females is costly. Alloparental care is of limited benefit to the dominant pair [16] and unrelated females may compete with related females for future dominance and mating opportunities.

Since a lack of affiliative behavior is associated with female red fox dispersal [34], unrelated females may opt to disperse rather than be evicted. Breeding by subordinate females is opportunistic [6], so there is a low probability that females with subordinate mothers will be closely related to future resident offspring. Hence remaining to provide alloparental care will not increase their fitness. Moreover, since dominants have a much longer life span than subordinates at high densities [16], philopatric females have a relatively low chance of territory inheritance and dispersal provides a greater chance of attaining dominance [27]. Thus daughters of subordinate females appeared to disperse voluntarily because there was a low probability of territory inheritance and limited indirect fitness benefits of remaining as a subordinate on their natal territory at both high and low densities.

Whilst this is the first study to demonstrate an effect of direct relatedness to the dominant female on offspring dispersal, several recent studies have highlighted the importance of a range of maternal factors in influencing dispersal behavior in vertebrates. For example, prolonged prenatal exposure to maternal stress levels resulted in extended philopatry in the common lizard, *Zootoca vivipara* [35]; offspring dispersal behaviors in great tits (*Parus major*) vary in response to maternal parasitism by differential transfer of maternal yolk androgens [36]; mothers regulate offspring dispersal in western bluebirds (*Sialia mexicana*) through egg-laying order in response to environmental conditions [37]; and maternal social dominance in spotted hyenas (*Crocuta crocuta*) gives dispersing males a fitness advantage through faster growth and immigration into stronger clans [38].

### Conclusions

Relatedness to resident females is important in juvenile dispersal in group-living mammals: female offspring unrelated to, and male offspring related to, the dominant female disperse. Paternity had no effect on dispersal of either sex. Male dispersal may be driven by inbreeding avoidance, whereas female dispersal appears to be influenced by the fitness advantages associated with residing with the same-sex dominant parent. Selection pressure on paternal control of offspring dispersal was low due to the limited costs associated with retaining unrelated males and the need for alternative inbreeding avoidance mechanisms between the dominant male and his female offspring. These findings have important implications for the evolution of dispersal and group living in social mammals, and our understanding of a key biological process.
